# On Syncope Senilis from Gastric Irritation

**Published:** 1856-07

**Authors:** 


*On Syncope Senilis from Gastric Irritation.* By JOHN HIGGINBOTTOM, F. R. S., F. R. C. S.

I have given the name of "syncope senilis" to this affection, particularly to direct the attention of the profession to the aged. The same complaint is common to all ages, but in a more aggravated form in infancy and old age. I am not aware that the affection has been specially noticed by any author, except under the head of indigestion, and the sufferers themselves often call it a bilious attack. I do not think that the symptom of syncope is so apparent in infancy; and I believe in middle age the attacks are slighter, and not often serious. The syncope in old age is very apparent, and is the first symptom requiring prompt attention, for if remedies are neglected, the complaint becomes sometimes much aggravated, and is followed by convulsion and death.

It is about thirty years since I first noticed particularly the syncope senilis. The subject was about 70 years of age. I thought at that time it was a precursor of an attack of apoplexy, the patient having had a slight paralysis when about 23 years of age, which affected him slightly through life. I was glad to find on his recovery, that there was no increase of his paralytic symptoms. Since that time, I have often observed the same syncope, unattended by any permanent ill effects.

My patients have been from 68 to 86 years of age; the youngest 68, and the oldest 86. I am not aware that they have labored under any organic disease whatever; but we all know, that at an advanced age the brain and heart, the nervous and vascular system, are frequently more inactive, and in an impaired condition.

In the cases I have attended of syncope senilis, gastric irritation appears to have been the sole cause of the attack. At that advanced age, mastication is very imperfectly or not at all performed, for want of teeth; solid animal food has been eaten when the stomach has been in an unfit state to assimilate it, usually after having had a longer walk than the patient has been accustomed to, or had more muscular exertion than usual, so as to produce fatigue, and sometimes after exposure to cold; all which tend to weaken the power of the stomach. On this account the food remains an indigestible mass in the stomach, and gives rise to gastric irritation, producing syncope and convulsion, which sometimes follows, often slight at first, but becoming more formidable, or even fatal, if proper remedies are not promptly used.

I was called to a patient about three o'clock in the morning, his wife having been awoke by his hard breathing and noise in his throat. She found her husband was in a fit. I was directly sent for. When I arrived he had partially recovered, but very soon after he had a second fit, which had the appearance of a slight attack of epilepsy, attended with convulsion, but had no bitten tongue, as is usual in severe attacks of epilepsy. As soon as he was sufficiently recovered from the attack, so that he could swallow, I gave him half a drachm of the powder of ipecacuanha with fifteen grains of the bicarbonate of potass, which was followed by full vomiting; he ejected lumps of solid beef, which appeared to have been swallowed, or rather bolted, without having been masticated at all; one of the pieces, I observed, was about an inch long and three quarters of an inch in thickness. Although the food had been taken into the stomach about sixteen hours, the acute corners and edges of the beef appeared as if just cut with a sharp knife, not the least digested. No further remedy was required after the emetic, but attention to the bowels, which he reluctantly submitted to, saying he was quite well.

In a month afterwards he had another fit of a similar nature. He fell down in a moment on the floor, and remained in the same state as in the former case for half an hour; the same remedies were resorted to as before, and he recovered quickly. I expect the patient will have a return of the syncope, as he is very willful, and will not attend to any means of prevention. This patient was the youngest, being 68 years of age. Previous to the first fit he had been using much muscular exertion, still being active in business.

Another case is that of an old patient of 86 years, who at intervals of a few weeks had several similar attacks of syncope. After the last fit, attended with slight convulsion, I was induced to think it had been occasioned by taking solid food, which was swallowed after imperfect mastication; on that account I forbade him the use of animal food altogether. This regimen he has now strictly adhered to for some months, except a few times having taken a small quantity of tripe. He has had no return of his fainting fit, a much longer time having now elapsed than the interval, after which he had several of the previous attacks. I would make an observation here, as a contrast to the former case I have related in the younger man, that at a more advanced age the patient does not recover so quickly from the attack, but requires particular attention to the digestive organs for some days with gentle aperients, and saline medicine in a state of effervescence.

It is not unusual for even young men to have similar attacks from indigestion, when sudden syncope for a short period comes on, recovery taking place in a few moments. The same attack at an advanced age, I presume, would be attended with aggravated symptoms, such as those I have witnessed.

The lamentable illness and death of the Duke of Wellington appears to me to have been a case of "syncope senilis," which became aggravated, and terminated fatally. In the "Life of the Duke of Wellington," by Stocqueler, it is stated that "the health of his Grace had been unusually good for some days, and on Monday, the 13th of September, it was remarked that he took a longer walk than usual through the grounds attached to the Castle." The *Lancet* of the 16th October, 1852, in the leading article says: "During some days preceding the 14th of September, 1852, the day of the Duke's death, there had been a hot midday sun, a considerable wind, chiefly from the North, and the evenings and nights were cold and chilly. The thermometer, on the night preceding the fatal event, was only six degrees above the freezing point; on the preceding day it had been up to ninety-two degrees Fahr. No precautions were taken to obviate the effect of such a change on the aged and necessarily weak system of the Duke, and the pallor of his countenance observed on the preceding Sunday showed that this influence was telling on the circulation. The stomach was ill prepared to receive a hearty dinner, and the difficulties of that organ were further increased by receiving a considerable quantity of food imperfectly masticated in consequence of the Duke's loss of teeth." "He took for dinner *mock turtle, turbot, venison*, and *pudding*." It is further added in the *Lancet:* "It is probable that had the Duke's stomach been relieved by vomiting in the early part of the morning, he would now be with us; it is even probable that such an effort, if successful at nine o'clock, might have saved him; but every hour added to the exhaustion, and rendered such an act difficult."

My brother-in-law, Dr. Marshal Hall, observes, in a paper in the *Lancet* of October 30th, 1852, "On the malady of the late Duke of Wellington,"—"It is obvious that if efficient vomiting could have been induced, the offending cause of this lamentable malady would have been removed, and all might have been well; he would, humanly speaking, still be with us. We have no evidence that the Duke of Wellington had any organic disease of either the brain or the heart. It is to be regretted that there was no post-mortem examination." I fully concur with the leading article in the *Lancet*, and with Dr. Marshal Hall's opinion, that an efficient vomiting at an early period would have been a most effectual remedy.

I know no emetic equal in such a case to half a drachm of the powder of ipecacuanha, with the addition of ten or fifteen grains of the bicarbonate of potass, as it corrects any acidity in the stomach, and produces full vomiting both safely and quickly; it has also the power of raising the system to its normal condition, without producing any unnatural excitement, and promotes the healthy secretions of the various organs of the body. The nausea and inefficient vomiting arising from natural efforts to empty the stomach, I have no doubt produces debility and exhaustion, when a full vomiting from ipecacuanha has the contrary effect. Should the first half-drachm of ipecacuanha not operate, a second such dose may be given with the greatest safety, it only having the effect of a more speedy operation. If vomiting still should not follow, the fauces might be irritated with a feather to excite it. I have for the last forty years given ipecacuanha emetics with the same freedom as I have purgatives, and never saw any bad result.

It might be thought by some individuals that abstaining from animal food at the period of old age might be attended with the loss of health and strength. I had an instance in a relation of my own family, who, at 70 years of age, quite abstained from animal food, and also from wine. After the lapse of ten years, when at the age of 80, he was requested by his relatives to resume his animal food and wine, he excused himself from taking either of them by saying he did not want them, for he was very healthy, and in good spirits, although very thin in body. He lived till he was nearly 90 years of age. This old gentleman, I apprehend, would have been a likely subject for the syncope senilis had he been in the habit of taking solid animal food, which he could not masticate, and would most probably have shortened his days.

At an advanced age when the physical powers of the body are declining, and second childhood approaching, and at that period when comparatively little exercise only can be taken, the body does not require the same solid food. Nature points out the use of milk and light farinacious matter as an aliment, as being more natural, and adapted to that period of life; such food alone is sufficient to keep the body in a healthy, cheerful, and happy state. It has been erroneously stated that "wine is the milk of old age;" I believe the truth is, that milk is the wine of old age, for both the first and second childhood, the most natural and the most nutritious. Dr. Erasmus Darwin used to say, "Milk is white blood." The oldest individuals I have known have lived principally upon milk diet. Second childhood may be treated much in the way as directed by the late Dr. James Hamilton, professor of midwifery in the University of Edinburgh: "Plenty of milk, plenty of flannel, and plenty of sleep or rest."—*Lancet, from Am. Med. Monthly.*

"*Syocope senilis*" will doubtless have a run amongst those who carry their knowledge on the tips of their tongues; but if it occurs "in a more aggravated form in *infancy* and old age," and if "it is not usual for even young men to have similar attacks," how can it hold its place? Then, if it has not been noticed by any author "except under the head of indigestion," what matter? for under the head of indigestion, there is ample room for such inquiry. But suppose "gastric irritation" should be the effect and not the "sole cause," and that the food remained undigested because the brain was at fault? Be this as it may, it is well to empty the stomach; but is an emetic safe with cerebral congestion? Moreover, as to the merits of milk, is not milk converted into curd in the stomach, and is not curd found rather indigestible in some cases? The writer of this article may be right, but it may be well to hint dissent, when sanguine people are prone to beg the question.—*Ed. Dub. Press, from Am. Med. Monthly.*

